# Maternal Obesity Induced by Diet in Rats Permanently Influences Central Processes Regulating Food Intake in Offspring

**DOI:** 10.1371/journal.pone.0005870

**Published:** 2009-06-11

**Authors:** Shona L. Kirk, Anne-Maj Samuelsson, Marco Argenton, Hannah Dhonye, Theodosis Kalamatianos, Lucilla Poston, Paul D. Taylor, Clive W. Coen

**Affiliations:** Division of Reproduction and Endocrinology, King's College London, London, United Kingdom; Pennsylvania State University, United States of America

## Abstract

Hypothalamic systems which regulate appetite may be permanently modified during early development. We have previously reported hyperphagia and increased adiposity in the adult offspring of rodents fed an obesogenic diet prior to and throughout pregnancy and lactation. We now report that offspring of obese (OffOb) rats display an amplified and prolonged neonatal leptin surge, which is accompanied by elevated leptin mRNA expression in their abdominal white adipose tissue. At postnatal Day 30, before the onset of hyperphagia in these animals, serum leptin is normal, but leptin-induced appetite suppression and phosphorylation of STAT3 in the arcuate nucleus (ARC) are attenuated; the level of AgRP-immunoreactivity in the hypothalamic paraventricular nucleus (PVH), which derives from neurones in the ARC and is developmentally dependent on leptin, is also diminished. We hypothesise that prolonged release of abnormally high levels of leptin by neonatal OffOb rats leads to leptin resistance and permanently affects hypothalamic functions involving the ARC and PVH. Such effects may underlie the developmental programming of hyperphagia and obesity in these rats.

## Introduction

The developmental overnutrition hypothesis suggests that maternal obesity and/or gestational diabetes in humans may predispose offspring to altered energy balance and increased adiposity in adulthood [1]–[4]. This hypothesis has gained strength with recent reports of an association between excessive weight gain in pregnancy and the BMI of the adolescent child and of a greater influence of maternal BMI than paternal BMI on offspring adiposity [5]–[7]. Animal models have proven invaluable in understanding developmental programming of adult disease. We have recently reported that offspring of mice or rats in which obesity had been induced by prolonged consumption of an obesogenic diet display hyperphagia, increased fat mass and hyperleptinaemia in adulthood [8], [9]. Other studies on experimental animals indicate that nutritional imbalance during pregnancy and lactation may lead to permanent modification of food intake due to developmental plasticity in the hypothalamus [10]–[18].

Studies on genetically hyperphagic (*ob/ob*) mice have demonstrated a neurotrophic role for leptin in the development of projections from the arcuate nucleus (ARC) to the hypothalamic paraventricular (PVH) nucleus [19]. The timing and magnitude of the neonatal leptin surge, normally present during the second postnatal week in rodents [20], can be perturbed by maternal undernutrition, thereby altering hypothalamic development, with persistent effects on energy balance [21]–[23]. In contrast, the present study addresses the hypothesis that a maternal calorie-rich diet and consequent obesity lead to hyperphagia in adult offspring through processes involving impaired leptin-signalling and altered neuronal development. We have characterised the neonatal profiles of serum leptin and adipose leptin mRNA and the composition of ingested milk in the offspring of obese dams (OffOb rats). To our knowledge, this is the first study to investigate the effects of maternal obesity on the neonatal leptin surge. We have also assessed behavioural and cell-signalling responses to exogenous leptin and the density of immunoreactivity for orexigenic and anorexigenic peptides in the hypothalamic paraventricular nucleus (PVH) prior to the onset of hyperphagia. This study thereby investigates the processes underlying the non-genetic transmission of an obesogenic trait from mother to offspring.

## Results

### Development of Maternal Obesity

**Figure 1:**
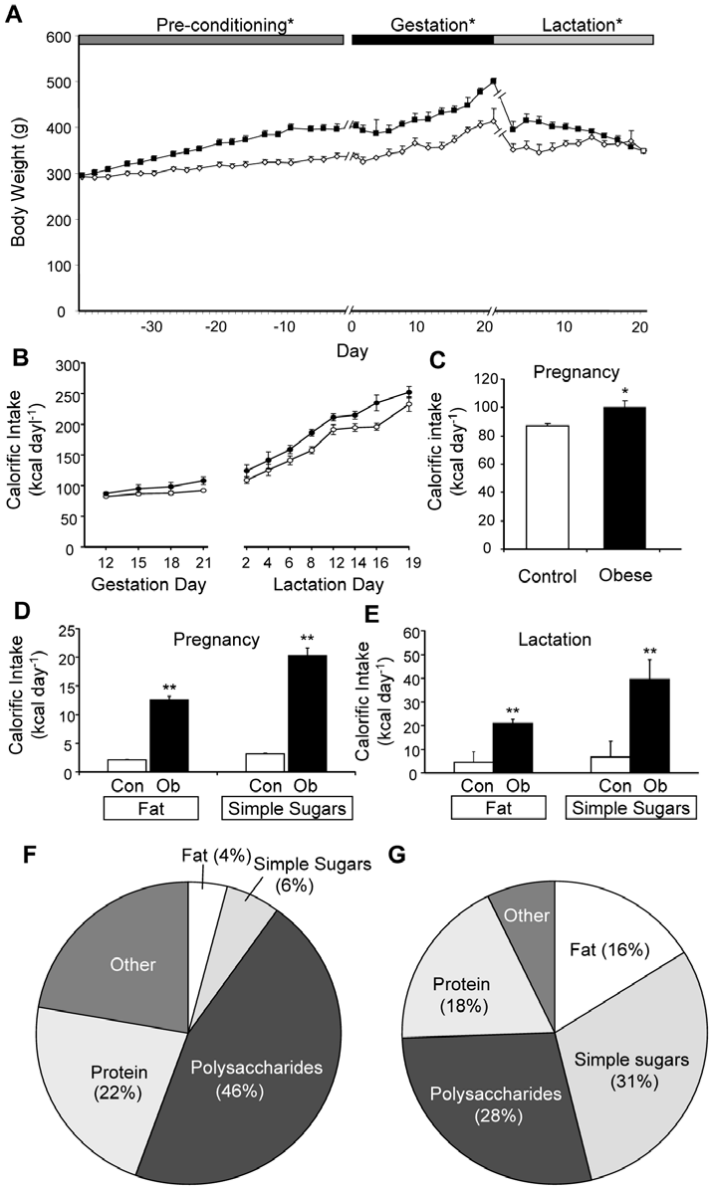
Maternal body weight and food intake in rats fed control or obesogenic diet. Body weight (A) was recorded for 6 weeks prior to pregnancy and throughout pregnancy and lactation for the animals on the control (open symbols) or obesogenic (closed symbols) diet; calorific intake was recorded throughout pregnancy and lactation (B). Average daily calorific intake from all sources during pregnancy (C) and average daily calorific intake from fat or simple sugars during pregnancy (D) or lactation (E) for the animals on the control (Con) or obesogenic (Ob) diet. Macronutrient content of ingested food (expressed as percentage by weight) for control (F) or obese (G) dams during lactation; “other” includes cellulose, ash, water etc. * p<0.05 and ** p<0.01 *versus* control dams (n = 11–12).

Female Sprague Dawley rats consuming the highly palatable fat- and sugar-rich diet became significantly heavier than control animals after 10 days (Fig. 1A). After 6 weeks on this obesogenic diet, they were 20% heavier than controls, at which point they were mated. The weight difference was maintained throughout pregnancy by significantly increased calorific intake of both fat and simple sugars (Fig. 1A–D). During lactation, dams on the obesogenic diet continued to show a significantly higher calorific intake from fat and simple sugars than the control dams (Fig. 1E). The obese dams consumed approximately 4 times more fat and 5 times more simple sugars than the control dams during pregnancy and lactation (Fig. 1F,G; data presented for lactation; similar data for the pre-conditioning period and during pregnancy not shown).

### Post-weaning Body Weight and Fat Mass in Offspring of Control or Obese Dams

**Figure 2:**
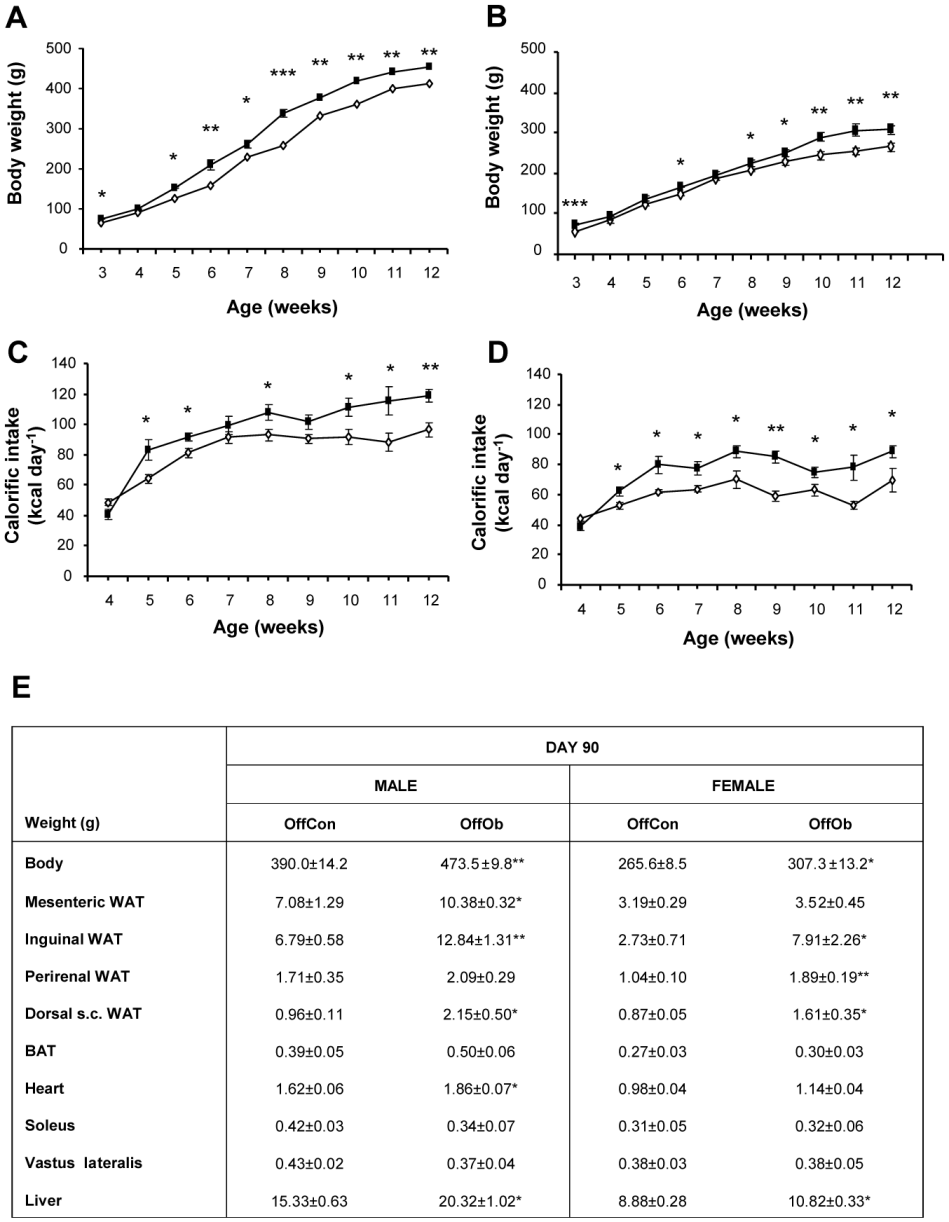
Post-weaning characteristics of offspring of control and obese dams. Body weight for males (A) and females (B) and calorific intake for males (C) and females (D) were recorded post-weaning for offspring of control (open symbols) or obese (closed symbols) dams. Offspring body and tissue weights were recorded at postnatal Day 90 (E). OffCon = offspring of control dams; OffOb = offspring of obese dams; WAT = white adipose tissue; BAT = brown adipose tissue; s.c. = subcutaneous. * p<0.05 and ** p<0.01 *versus* offspring of control dams (n = 8–11).

Male and female OffOb rats were heavier at weaning than offspring of control dams (OffCon rats; Fig. 2A,B); however, by 30 days of age, after weaning onto standard chow, body weight was similar between the two groups (body weight [g]: OffCon males: 100.9±2.5 *versus* OffOb males 100.1±4.3 males; OffCon females 96.4±1.7 *versus* OffOb females 94.8±3.1). OffOb rats developed hyperphagia from 5–6 weeks of age, showing a significant increase in calorific intake and body weight, which persisted into adulthood (Fig. 2A–D). At 90 days of age, OffOb rats weighed more than OffCon rats (body weight [g]: OffCon males 390.0±14.2 *versus* OffOb males 473.5±9.8 males p<0.01; OffCon females 265.6±8.5 *versus* OffOb females 307.3±13.2 p<0.05) and had markedly greater fat mass than OffCon rats in the inguinal, dorsal (interscapular) subcutaneous, mesenteric (males only) and perirenal (females only) fat pads (Fig. 2E). Liver weight was significantly greater in OffOb males and females; hearts were heavier only in OffOb males (Fig. 2E). Weights of brown adipose tissue and *soleus* and *vastus lateralis* muscles were similar in OffOb and OffCon animals (Fig. 2E).

### Maternal Obesogenic Diet Leads to Altered Parameters of Metabolic Function in Neonatal Offspring

**Figure 3:**
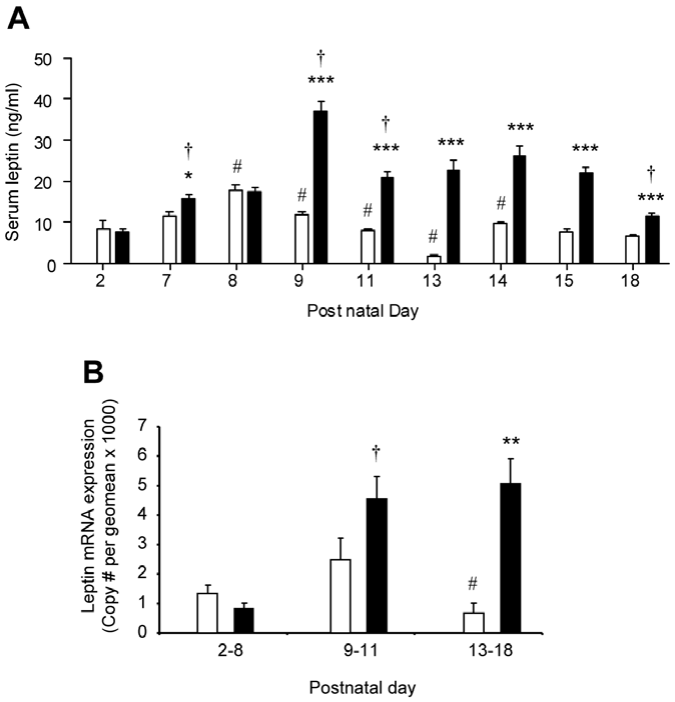
Neonatal serum leptin concentrations and adipose leptin mRNA expression in offspring of control and obese dams. Serum leptin was measured in offspring of control dams (open bars) and obese dams (closed bars) on postnatal days 2, 7, 8, 9, 11, 13, 14, 15 and 18 (A). Leptin mRNA expression in abdominal fat is presented for postnatal days 2–8, 9–11 and 13–18 (B). * p<0.05, ** p<0.01 and *** p<0.01 *versus* offspring of control dams for the same period (n = 3–6). For longitudinal comparisons, a significant difference (p<0.05) from the preceding period is indicated by # for offspring of control dams and by † for offspring of obese dams.

**Figure 4:**
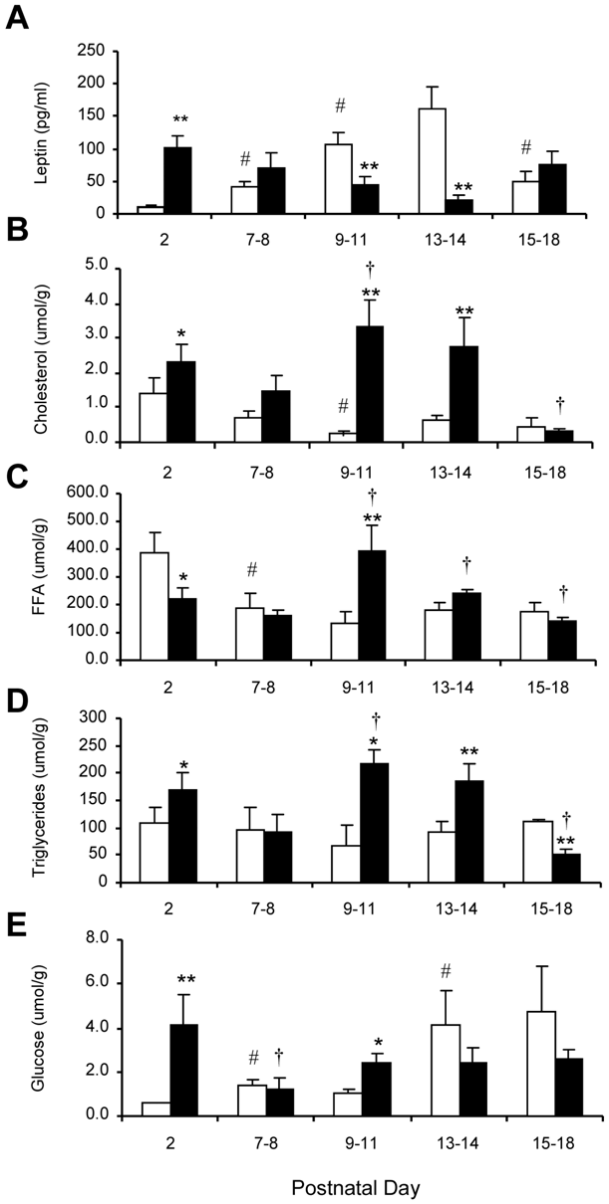
Constituents of milk from control and obese dams by analysis of pups' stomach contents. The concentration of leptin (A), cholesterol (B), free fatty acids (FFA) (C), triglycerides (D) and glucose (E) was assayed in stomach contents (as a proxy measure of the dams' milk content) from offspring of control dams (open bars; n = 4–8) and obese dams (closed bars; n = 4–8) throughout suckling. * p<0.05 and ** p<0.01 *versus* offspring of control dams at the same period. For longitudinal comparisons, a significant difference (p<0.05) from the preceding period is indicated by # for offspring of control dams and by † for offspring of obese dams.

Given the putative role of the rodent neonatal leptin surge in persistent effects on offspring energy balance, we investigated leptin profiles in pup serum and stomach contents (as a proxy measure of the dam's milk content) over the suckling period. The serum leptin profile, which typically shows a surge during the neonatal period [21]–[23] was greatly amplified and prolonged in OffOb rats (Fig. 3A). In OffCon rats the leptin surge showed two peaks: an initial peak at postnatal Day 8 and a second smaller peak at Day 14. In contrast, the leptin surge in OffOb rats remained elevated throughout the latter period of lactation, being significantly higher than in OffCon rats on postnatal days 7, 9, 11, 13, 14, 15 and 18 (Fig. 3A). The leptin mRNA profile in pup abdominal white adipose tissue sampled from birth to weaning showed significantly greater expression in OffOb rats during the extended period of their leptin surge (Fig. 3B). This suggests that the pup's adipocytes are the principal source of the amplified and prolonged serum leptin surge. The possibility that the leptin concentration in the dam's milk, as estimated from the stomach contents of the pups, contributed to the serum leptin profile was investigated; however, the mismatch between the ingested and serum leptin profiles in OffOB and OffCon rats failed to support this hypothesis (Fig. 4A). Furthermore, the leptin concentration in the stomach contents was at least two orders of magnitude lower than in the pup's serum. Nevertheless, the profiles of ingested cholesterol, free fatty acids, triglycerides and glucose in OffOb rats showed a marked rise on postnatal days 9–11 (Fig. 4B–E); this coincided with the onset of sustained elevated serum leptin (Fig. 3A). Analysis of the fatty acid profiles in the milk during the leptin surge (Days 9–18) showed an increase in the ratio of arachidonic acid (n-6) to eicosapentaenoic and docosahexaenoic acids (n-3): n-6∶n-3 ratio [mean±SD]: OffCon 1.71±0.54 vs. OffOb 3.85±1.59, P<0.01.

### Leptin Resistance in Offspring of Obese Dams

**Figure 5:**
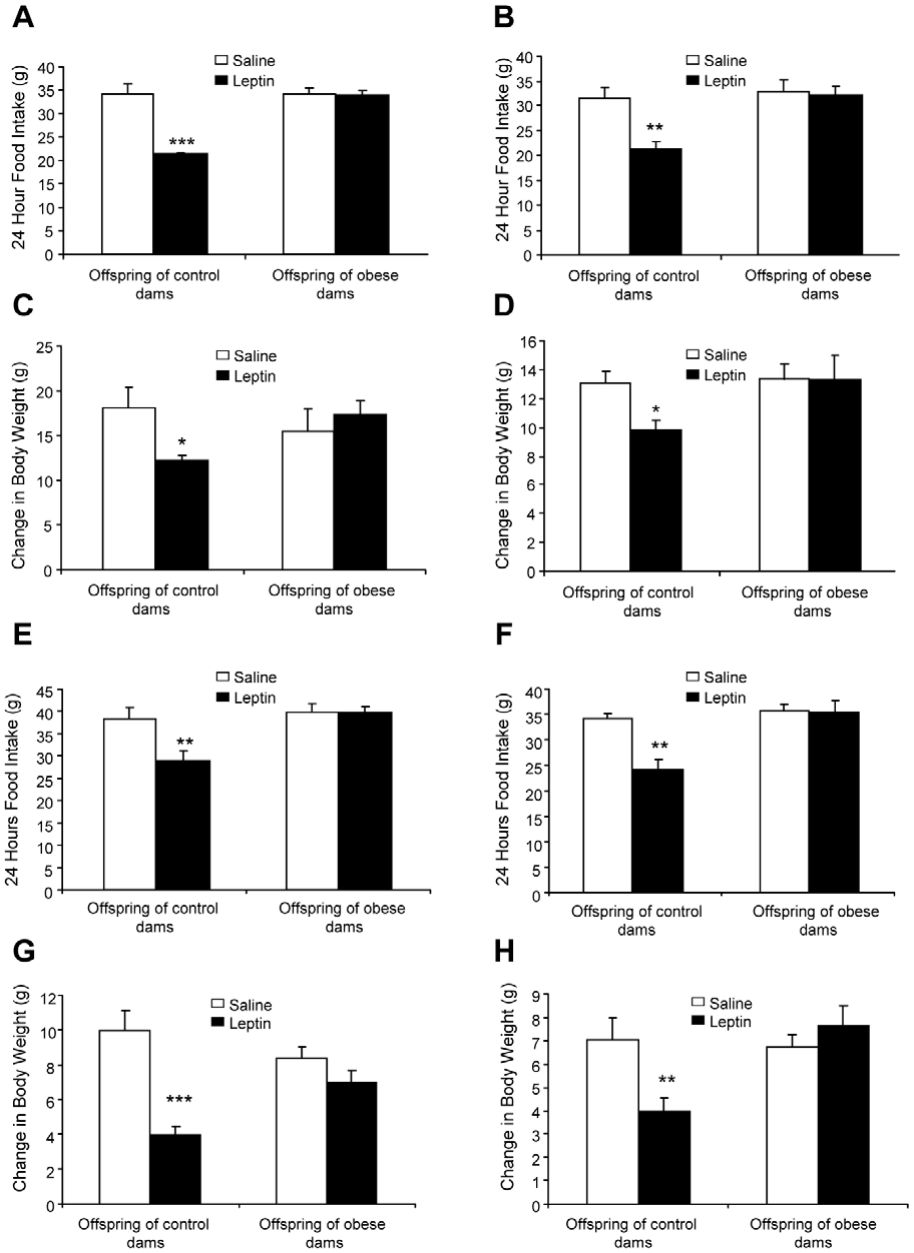
Behavioural responses to leptin in juvenile and adult offspring of control and obese dams. Food intake for males (A) and females (B) and change in body weight for males (C) and females (D) recorded over 24 hours following administration of leptin (10 mg/kg, i.p.) in 30 day-old offspring of control or obese dams. Food intake for males (E) and females (F) and change in body weight for males (G) and females (H) in 90 day-old offspring of control or obese dams. OffCon = offspring of control dams; OffOb = offspring of obese dams; * p<0.05 and ** p<0.01 ***p<0.001, *versus* offspring of control dams (n = 6).

The possibility that OffOb rats are leptin resistant was investigated at Day 30, when endogenous circulating levels of leptin showed no significant differences between OffOb and OffCon rats (leptin [ng/ml]: OffCon males: 3.5±0.5 *versus* OffOb males 2.8±0.4 males; OffCon females 3.6±0.3 *versus* OffOb females 3.0±0.7). Leptin (10 mg/kg) administered intraperitoneally failed to reduce food intake and body weight over a 24-hour period in male and female OffOb rats compared with OffCon rats (Fig. 5A–D). This resistance to the appetite- and weight-reducing actions of leptin was also apparent at Day 90 (Fig. 5E–H), when the serum leptin concentration was significantly higher in OffOb rats (leptin [ng/ml]: OffOb males 21.74±1.84 *versus* OffCon males 15.68±0.73, P<0.01; OffOb females 10.71±1.23 *versus* OffCon females 7.92±0.90, P<0.05).

**Figure 6:**
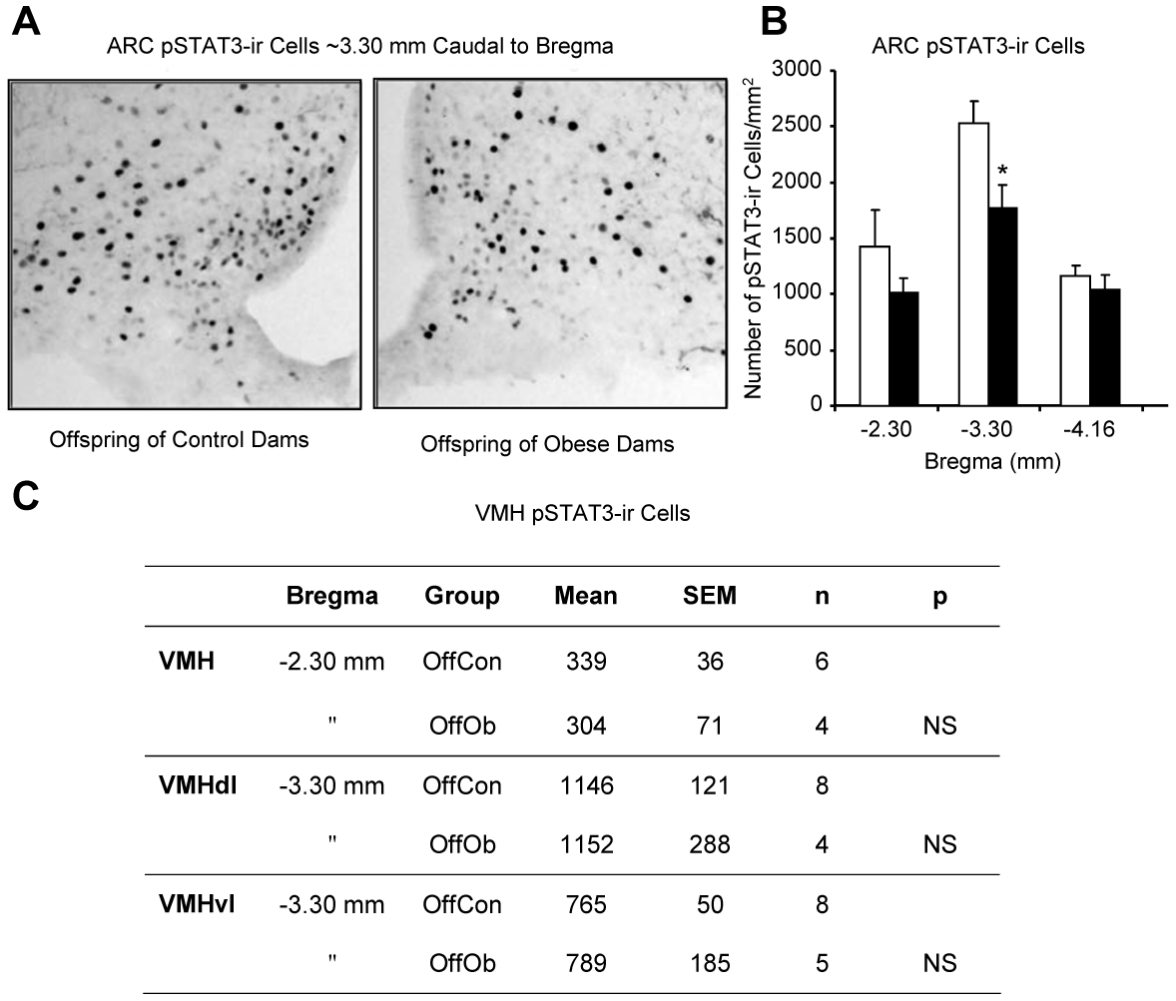
Signalling responses to leptin in juvenile and adult offspring of control and obese dams. Representative images (A) and quantitative analysis (B and C) of leptin-induced pSTAT3-immunoreactive (ir) cells (per mm^2^) in the arcuate nucleus (ARC) and ventromedial hypothalamic nucleus (VMH). Leptin (10 mg/kg, i.p.) was administered 45 min prior to administration of anaesthetic and perfusion fixation of the brain. OffCon = offspring of control dams; OffOb = offspring of obese dams; dl = dorsolateral; vl = ventrolateral. * p<0.05 *versus* offspring of control dams (n = 4–8).

The processes underlying the behavioural evidence for leptin resistance were investigated by determining the number of cells immunoreactive for pSTAT3, which is induced following activation of leptin receptors [24], in the ARC and ventromedial hypothalamic nucleus (VMH) in response to exogenous leptin (10 mg/kg, i.p.). In the ARC, at approximately 3.30 mm caudal to bregma, OffOb rats displayed fewer pSTAT3-immunoreactive neurons than OffCon rats (Fig. 6 A,B). No evidence of impaired leptin-signalling was found in the VMH (Fig. 6G).

### Reduced AgRP Projections to the PVH in Offspring of Obese Dams

**Figure 7:**
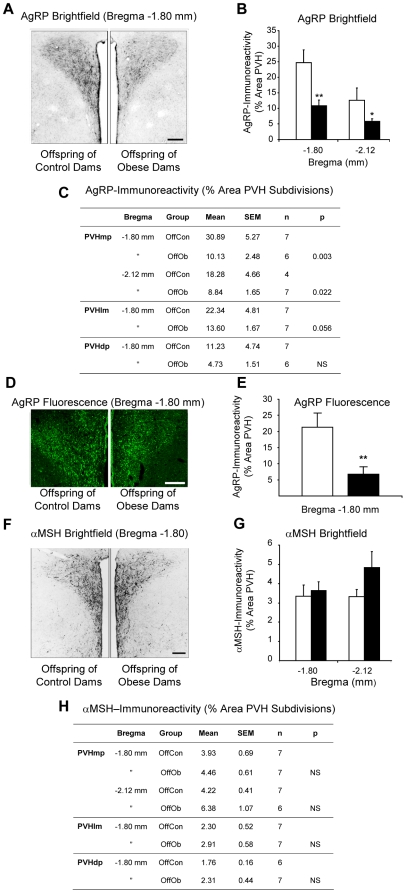
AgRP- and α-MSH-immunoreactivity in the PVH of offspring of control and obese dams. Representative brightfield images (A) and quantitative comparisons (B,C) of AgRP-immunoreactivity in the paraventricular hypothalamic nucleus (PVH) and its subdivisions in male offspring of control and obese dams. Representative confocal images (D) and quantitative comparisons (D,E) of AgRP immunofluorescence in the PVH of female offspring of control and obese dams. Representative brightfield images (F) and quantitative comparisons (G,H) of α-MSH-immunoreactivity in the PVH and its subdivisions in male offspring of control and obese dams. OffCon = offspring of control dams (open bars); OffOb = offspring of obese dams (closed bars); mp = medial parvocellular; lm = lateral magnocellular; dp = dorsal parvocellular. * = p<0.05 and ** = p<0.01 *versus* control. Scale bars = 200 µm.

The behavioural and cellular leptin resistance at Day 30 in OffOb rats (Fig. 5 and 6) is preceded by an amplified and prolonged leptin surge during the pre-weaning phase (Fig. 3A). We hypothesise that this extended and abnormally high surge is responsible for down-regulation of leptin-signalling in the neonatal period. Since leptin has neurotrophic actions on the development of projections from the ARC [19], such down-regulation may impair the normal development of those projections. Neurons synthesising agouti-related peptide (AgRP) are restricted to the ARC and contain the orexigenic neuropeptide Y [25], [26]; consequently all AgRP-immunoreactivity detected in the PVH derives from neurones in the ARC. A separate neuronal population in the ARC expresses the precursor for α-melanocyte stimulating hormone (α-MSH) and sends projections to the PVH [27]; it is not clear whether this is the sole source of α-MSH in the PVH. The α-MSH precursor is also expressed in a subpopulation of neurons within the nucleus of the solitary tract, a nucleus with extensive projections to the PVH [28]; there is, however, doubt about whether those neurons project to the hypothalamus [29], [30]. At postnatal Day 30, the density of AgRP-immunoreactivity in the PVH (at approximately 1.80 mm and 2.12 mm caudal to bregma) was reduced in male and female OffOb rats (Fig. 7A–E). On analysis of PVH subdivisions, a statistically significant reduction in AgRP-immunoreactivity was reached only within the medial parvocellular region of the PVH (Fig. 7C). In contrast, no change in α-MSH-immunoreactivity was observed in the PVH (Fig. 7F–H).

## Discussion

The results of this study add to the evidence that nutritional status early in life can modify energy homeostasis in later life [1]–[4], [14], [31], [32]. The ‘developmental origins of adult disease’ hypothesis, which originally focused on the deleterious effects of maternal and fetal undernutrition [31], now encompasses the theory that maternal and fetal overnutrition is similarly disadvantageous [32]–[44]. Recently we have demonstrated that the adult progeny of mice or rats made obese by a high-fat/sugar diet become hyperphagic and obese on a standard chow diet [8], [9] furthermore, they develop insulin resistance, hyperleptinaemia and hypertension [8]. The present findings in rats suggest that leptin resistance acquired by the animal early in postnatal life plays a critical role in the aetiology of the phenotype induced by maternal overnutrition.

### Central Leptin Resistance in Juvenile and Adult Offspring of Obese Rats

Leptin plays a key role in energy balance through inhibition of orexigenic systems and stimulation of anorexigenic systems in the ARC [45]–[48]. Leptin resistance is generally associated with a state of chronic obesity in which hyperleptinaemia fails to maintain lean body weight set points by inhibiting food intake [48]–[52]. In the present study, juvenile OffOb rats (Day 30) showed a loss of the inhibitory effects of leptin on food intake and body weight, at a time at which serum leptin was not raised; this resistance to leptin was still present in adulthood (Day 90), when the animals were obese and hyperleptinaemic. Rats with a genetic susceptibility to diet-induced obesity (DIO) also display leptin resistance prior to development of the obese phenotype [53], [54]. In the ARC, VMH and DMH of pre-obese DIO rats there are reductions in the expression of leptin receptor (LRb) mRNA, leptin binding and leptin-signalling [53]–[55]. In OffOb rats, reduced phosphorylation of STAT3 was restricted to the ARC. As also observed in DIO rats [56], the OffOb animals showed an attenuation of AgRP-immunoreactivity in the PVH which is derived from neurones the ARC. In the DIO strain, the imposition of maternal overnutrition during pregnancy failed to enhance the loss of projections from the ARC to the PVH; thus, the genetic background seems to prevail in that model [56].

Hyperleptinaemia induced exogenously in normal rats during the first 10 days of postnatal life leads to a permanent loss of the anorexic response to leptin [57], [58]. The processes which lead to central leptin resistance secondary to transient or chronic hyperleptinaemia remain poorly understood, although signalling molecules downstream of the leptin receptor are implicated [59]. A major finding of the present study provides cell-signalling and behavioural evidence of developmentally induced leptin resistance prior to the onset of chronic obesity and hyperleptinaemia. Bouret and colleagues (2008) observed decreased leptin-signalling during early postnatal development in the DIO model[56]. In comparison with the diet-resistant animals, DIO rats showed a 24% reduction in the number of ARC neurons with leptin-induced pSTAT3-immunoreactivity on Day 10 [56]. The results of that study are in accord with the present observation of reduced leptin-signalling in the ARC of juvenile OffOb rats. Thus, impaired leptin-signalling in pre-obese animals is a common feature of these genetically and epigenetically “programmed” models.

Given that leptin was administered peripherally in the present study, a transport defect may have contributed to the observed leptin resistance in the OffOb animals. However, impaired leptin-signalling is also observed in pre-obese DIO rats despite normal leptin transport across the blood-brain barrier [55]. Thus, although severely obese mice with chronic hyperleptinaemia, after 20 weeks on a high-fat diet, show reduced leptin transport across the blood-brain barrier [60], it seems unlikely that a transport defect accounted for the leptin resistance in the juvenile OffOb rats, which were not yet obese and had a normal level of serum leptin.

### Reduced AgRP-Immunoreactivity in the PVH

Juvenile OffOb rats showed a reduction in AgRP-immunoreactivity in the PVH, particularly within its medial parvocellular region. Since AgRP is synthesised only in the ARC [25], [26], this finding may reflect reduced synthesis/transport or increased release of the peptide and/or disrupted development of the AgRP-containing projections from the ARC to the PVH. A reduction in AgRP-immunoreactive fibres has been observed in leptin-deficient (*ob/ob*) obese mice [19] and in the DIO strain of rats [56]. Furthermore, ARC explants obtained from DIO rats during the early postnatal period show reduced neurite extension in response to leptin [56]. The absence of a significant change in α-MSH-immunoreactivity in the PVH of OffOb rats is also consistent with the DIO model [56].

It is not clear by what mechanisms a reduction in immunoreactivity for the orexigenic peptide AgRP in the PVH of *ob/ob*, DIO and OffOb rodents might be associated with their hyperphagia. As a possible explanation for this apparent paradox, it has been proposed that AgRP processes proliferate within the ARC and inhibit the anorexigenic α-MSH neurons [61]. Alternatively, it possible that AgRP-immunoreactivity in the PVH is depleted due to increased local release of this peptide, albeit from a reduced number of processes; if the net effect were orexigenic, the apparent paradox would be resolved.

### Significance and Source of the Amplified and Prolonged Neonatal Leptin Surge in OffOb Rats

A leptin surge normally occurs during the second postnatal week in rodents [20]. During that period, exogenous leptin does not suppress food intake in rats or mice [62]–[64]. Leptin's subsequent influence on food intake depends on developmental processes, which may be regulated by leptin itself at a critical concentration over a critical period. Several reports indicate that the neonatal leptin surge is disturbed in its timing and/or magnitude by maternal undernutrition, thereby altering hypothalamic development and inducing persistent effects on energy balance [21]–[23]. Hyperleptinaemia induced exogenously in rats during the first 10 days of postnatal life leads to reduced hypothalamic expression of leptin receptors [57]–[64]. We therefore hypothesise that the amplified and prolonged neonatal leptin surge in OffOb rats causes a similar down-regulation, leading to inhibition of leptin's neurotrophic actions and permanent leptin resistance. This is supported by the discovery that leptin administration to mice neonatally results in leptin resistance in adulthood [22]. Moreover, Vickers and colleagues [65] have recently reported that neonatal leptin treatment of rats from normally nourished dams results in increased diet-induced weight gain in adulthood. These findings contrast with a previous report from the same authors [21] showing that leptin treatment over a similar postnatal period can prevent offspring obesity associated with maternal undernutrition. Such divergent responses highlight the significance of maternal nutritional status in modulating the consequences of early life exposure to leptin.

The present findings provide insight into the origins of the altered serum leptin surge in the OffOb rats. Since leptin ingested by neonatal rats passes unchanged into the circulation [66], we initially hypothesised that milk-borne leptin ingested from an obese dam might provide a link between maternal body composition and hypothalamic development. However, the amplified and prolonged leptin surge in neonatal OffOb rats was not paralleled by a rise in ingested leptin; furthermore, the leptin concentration in the stomach contents was at least two orders of magnitude lower than in the pup's serum. Our observation that the OffOb rats' extended leptin surge was accompanied by elevated leptin mRNA expression in adipose tissue suggests that the source of the serum leptin was the pup's adipocytes rather than the dam's milk.

Although leptin ingestion by OffOb rats does not appear to be a significant factor in this experimental model, it is well established that the diet of rat dams affects milk composition [67], [68]. The marked rise in the concentration of cholesterol, free fatty acids, triglycerides and glucose in the stomach contents of the OffOb rats on postnatal days 9–11 coincided with the onset of the sustained elevation in serum leptin. It is possible that one or more of these variables may contribute to the extended leptin surge. The increase in fatty acid ingestion may be significant, since fatty acids (particularly n-6) can promote differentiation of preadipocytes into mature adipocytes [69], which express leptin [20]. Analysis of the fatty acid profiles in milk during the amplified and prolonged leptin surge showed an increase in the ratio of arachidonic acid (n-6) to eicosapentaenoic and docosahexaenoic acids (n-3). The fatty acid content of the ingested milk may indirectly affect hypothalamic development through modulation of the leptin surge. This suggests that intervention with a high n-3∶n-6 ratio diet may have therapeutic potential. The possibility of direct effects of ingested fatty acids [70] or glucose [71] on hypothalamic gene expression and development should also be recognised. Recent data highlight the significance of the suckling period in the aetiology of hyperphagia. In a cross-fostering study, using our maternal overnutrition model mice [8], we have found (unpublished) that offspring of lean dams suckled by obese dams display adult hyperphagia.

### Conclusions

This study shows that maternal obesity induced by diet, prior to and throughout pregnancy and lactation, results in offspring with a hyperphagic and obese phenotype in adulthood. Before the onset of the adult phenotype, these animals show not only cell-signalling and behavioural evidence of leptin resistance, but also attenuated AgRP-immunoreactivity in the PVH. Neonatally they display an amplified and prolonged surge of leptin, which is accompanied by elevated leptin mRNA expression in adipose tissue. We hypothesise that prolonged release of abnormally high levels of leptin before weaning leads to permanently impaired leptin-signalling and a consequent reduction in leptin's neurotrophic effects, possibly due to down-regulation of leptin receptors. Such effects may underlie the subsequent development of hyperphagia and increased adiposity in this experimental model.

## Methods

### Animals and Diets

Female Sprague-Dawley rats (Banting & Kingman, Hull, UK) were housed individually under standard laboratory conditions on a 12 h light: dark cycle (lights on at 07:00) in a temperature-controlled environment at 21±2°C and humidity of 40–50%. The animals had *ad libitum* access to food and water. The experiments were carried out in accordance with the UK Animals (Scientific Procedures) Act, 1986. Animals were allowed to habituate to the animal unit for one week before initiation of experiments. Male Sprague-Dawley rats (Banting & Kingman) were used for breeding. The rats were fed either an obesogenic or a control diet (n = 12 per group). The obesogenic diet, provided for 6 weeks before mating and throughout pregnancy and lactation, consisted of a semi-synthetic energy-rich and highly palatable pelleted diet (20% animal lard, 10% simple sugars, 28% polysaccharide, 23% protein [w/w], energy 4.5 kcal/g, Special Dietary Services, Wittam, UK), supplemented with sweetened condensed milk (Nestlé, Vevey, Switzerland) which was fortified with 3.5% mineral mix and 1% vitamin mix [w/w] (AIN 93G, Special Diets Services). The macronutrient contents of the food ingested on the control diet or on the obesogenic diet are indicated (Fig. 1F,G). The condensed milk was presented separately from the pellets in a stainless steel coop cup attached to the side of the cage with a wire dish holder to prevent spillage. The control rats received the standard maintenance diet (RM1; Special Diets Services) until 10 days before mating, when they were given the standard breeding diet (RM3) until weaning. Pregnancy was established, within a week of cohabitation with a male, in 100% of the females on the control diet and in 83% of the females on the obesogenic diet. Average litter size was greater for the obese dams (mean litter size±SEM: 12.3±0.60, OffOb, *versus* 10.4±0.64, OffCon, p<0.05). Litter size was standardised to 8 pups (4 male, 4 female) 48 hours after birth. All offspring were weaned at Day 21 and subsequently fed RM1 diet *ad libitum*. One male and one female from each litter were then sacrificed for blood and tissue collection at Day 30 and Day 90; remaining littermates were used in other studies. Organs weights were recorded and serum stored at −80°C for future analysis. After weaning of their pups, dams were sacrificed following an overnight fast; blood was collected and serum stored at −80°C. Animals tested for anorexic responses to leptin at Day 30 were fasted during the preceding night; the leptin was administered at 10.00 h.

In a separate cohort of animals, litters of control and obese dams were killed at several postnatal stages from Day 2 to Day 18. At each time-point (Days 2, 7, 8, 9, 11, 13, 14, 15 and 18), litters were killed by decapitation between 0800 and 1100 h. Blood samples were collected (trunk blood) and abdominal fat pads and stomach content collected and stored at −80°C until analyzed.

### Assessing Anorexic Responses to a Leptin challenge in Young Offspring of Obese Dams

At 30 or 90 days of age, the rats were tested for food intake in response to either leptin or saline after being fasted for 18 hours. Recombinant rat leptin (PeproTech, Inc., Rocky hill, NJ, USA) was dissolved in saline vehicle (0.9% w/v) and given as a bolus injection at the dose of 10 mg/kg body weight i.p. After the intraperitoneal treatment, the animals were housed singly, and food intake was measured over the 24 hour post-challenge period. Change in body weight was also recorded over the same period (Δ weight in grams).

### Biochemical Assays

Neonatal stomach contents were used as an indirect measurement of the milk contents. Stomach contents were extracted in an equal volume of water, employing an ultrasonicator and centrifuged for 15 min. The supernatant was then used for glucose and leptin analysis. Remaining sample was further extracted in ethanol, shaken and centrifuged for 15 min. The concentrations of leptin (in stomach contents and serum) were measured by ELISA (RD291001200 kit; Biovendor, Modrice, Czech Republic); glucose, triglycerides, cholesterol and free fatty acids were determined by an autoanalyzer (Hitachi 912, Roche Diagnostics, Almere, The Netherlands) using commercial kits (Gluco-Quant/HK, Triglycerides-GPO-PAP, Cholesterol-CHOD-PAP from Roche Diagnostics, Brussels, Belgium; NEFA-C from Wako Chemicals, Neuss, Germany).

### Real-time PCR

Total RNA was extracted from abdominal fat from neonatal rats on Days 2, 7, 8, 9, 11, 13, 14, 15 and 18 by standard Trizol (Sigma-Aldrich Ltd., Poole, UK) method. RNA quantity and integrity were assessed by optical density using a Nanodrop-1000 spectrophotometer (NanoDrop Products, Wilmington, USA). Reverse transcription was carried out from 1 µg of RNA sample with QuantiTect Reverse Transcription Kit (cat. no. 205311; Qiagen, Crawley, UK) according to instructions; cDNA was stored at −80°C. Intron-spanning primers for leptin (accession number NM_013076) for real-time PCR were designed using Universal Probelibrary (Roche Diagnostics Ltd., Burgess Hill, UK); primers (F: 5′-CCA GGA TCA ATG ACA TTT CAC-3′ and R: 5′-AAT GAA GTC CAA ACC GGT GA-3′) were obtained from Operon Biotechnologies GmbH (Cologne, Germany). Two microlitres of cDNA was used in 10 µl amplification reactions, containing 5 µl SYBR green fluorescent dye (QuantiFast SYBR Green PCR Kit; Qiagen Crawley, UK), 0.5 µl 10 mM forward and reverse primers and 2 µl RNA-ase free water, with the following cycling conditions: initial activation for 5 minutes at 95°C, followed by 40 cycles of denaturation at 95°C for 10 seconds and combined annealing/extension at 60°C for 30 seconds. Sample copy numbers for leptin, 28S and β-actin were determined by standard curves (leptin, R^2^ = 0.997; 28S R^2^ = 0.996; β-actin R^2^ = 0.94) and used to calculate the concentrations of leptin mRNA relative to the geometric mean of 28S and β-actin mRNAs, with Rotorgene 6000 series software (Corbett Research, Mortlake, Australia).

### Immunohistochemistry

On postnatal Day 30, one male rat from each litter was given saline or leptin 45 minutes before anaesthesia (Pentoject, 50 mg/kg, i.p.; Animalcare Ltd., York, UK) and transcardiac perfusion with 4% paraformaldehyde. Coronal cryostat sections (30 µm) containing the ARC and VMH were processed for pSTAT3 immunohistochemistry according to a method modified from Levin and colleagues [55]. Floating sections were immersed in phosphate buffered saline (PBS) containing 1% NaOH and 1% hydrogen peroxide for 20 minutes and then in PBS containing 0.3% glycine for 10 minutes, followed by PBS containing 0.15% SDS for 10 minutes. After incubation in 2% donkey serum containing 0.4% Triton X-100 for 2 hours, the sections were immersed in monoclonal rabbit anti-pSTAT3 (9145L, 1∶2000; Cell Signaling Technologies Inc., Boston, MA, USA) for 7 days at 4°C. The sections were then incubated for 1 hour in biotinylated donkey anti-rabbit IgG (1∶1000; Stratech Scientific Ltd., Newmarket, UK) and subsequently in avidin-biotin-peroxidase complex (ABC, 1∶1000; Vector Laboratories, Peterborough, UK) for 1 hour. Immunoreactivity was detected by incubation in Tris buffer (pH 7.6) containing 0.05% 3,3′-diaminobenzidine-4HCl (DAB), 0.15% nickel ammonium sulphate (NAS) and 0.005% H_2_O_2_.

Additional untreated male and female OffCon and OffOb rats were perfused transcardially with 4% PFA on postnatal Day 30 to determine AgRP- and α-MSH-immunoreactivity in the PVH. Coronal cryostat sections (30 µm) were treated with 0.5% Triton X-100 for 30 minutes, 0.5% hydrogen peroxide for 15 minutes and 2% normal donkey serum for 1 hour. For brightfield immunohistochemistry, sections were then incubated for 4 days at 4°C in rabbit anti-AgRP (H-003-57, 1∶20,000; Phoenix Europe GmbH, Karlsruhe, Germany) or sheep anti-α-MSH (1∶50,000, AB5087; Millipore, Livingstone, UK). Following a 2-hour incubation in biotinylated donkey anti-rabbit IgG (1∶800; Stratech Scientific Ltd.) or biotinylated donkey anti-sheep IgG (1∶1000; Stratech Scientific Ltd.), sections were incubated in ABC (1∶2000; Vector Laboratories) for 90 minutes. Immunoreactivity was detected as for pSTAT3. For confocal immunohistochemistry, sections were incubated in rabbit anti-AgRP (H-003-57, 1∶4,000; Phoenix Europe GmbH) for 4 days at 4°C. They were subsequently incubated in biotinylated donkey anti-rabbit IgG (1∶1000; Stratech Scientific Ltd.) for 2 hours, followed by ABC (1∶3000; Vector Laboratories) for 90 minutes, before being exposed to biotinylated tyramide (1∶500; PerkinElmer Laboratories, Waltham, USA) for 30 minutes in the presence of 0.005% H_2_O_2_. The sections were then immersed in AlexaFluor 488-conjugated streptavidin (1∶500; Invitrogen Ltd., Paisley, UK) for 24 hours at 4°C. For these sections, coverslips were applied over an anti-fade reagent (Prolong; Invitrogen Ltd.).

### Image analysis

Following immunohistochemical visualisation of pSTAT3, AgRP or α-MSH, computerised image analysis, blind to treatment, was used to determine the number of pSTAT3-immunoreactive cells within the ARC and VMH and the density of AgRP- and α-MSH- immunoreactivity within the PVH. pSTAT3-immunoreactive cells in the ARC and VMH were counted (MCID software, version 7.0; Interfocus Imaging Ltd., Cambridge, UK) and expressed as cells/mm^2^. The mean number of pSTAT3-immunoreactive cells on each side in 3–6 sections from each rat, within approximately ±0.20 mm of the rostro-caudal level cited [72] was used for statistical analysis. The percentage of the area containing immunoreactivity for AgRP or α-MSH within the whole PVH or its subdivisions was quantified (MCID software, version 7.0); thresholding was optimised blind to treatment for each animal. For brightfield immunoreactivity, the mean immunoreactive density on each side in 3 sections from each rat, within approximately ±0.10 mm of the rostro-caudal level cited [72], was used for statistical analysis. Analysis of AgRP-immunofluorescence within the PVH was undertaken following collection of confocal images at 1 µm intervals (10–15 optical sections) through each section. For immunofluorescence, the mean immunoreactive density on each side in 2 sections from each rat, within approximately ±0.10 mm of the rostro-caudal level cited [72], was used for statistical analysis.

### Analysis of fatty acids

Fatty acid composition of milk extracted from pup stomach contents was analyzed by gas-liquid chromatography, using a modified version of the direct one step transesterification method by Lepage & Roy (1986)[73].

### Statistical analysis

Data are expressed as mean±SEM, unless stated, and compared using 2-way ANOVA followed by a *post hoc* Dunnett's test, or Student's *t* test. p≤0.05 was regarded as significant.
